# "Soccer player's heart": assessment of left ventricular adaptation by cardiac magnetic resonance imaging

**DOI:** 10.1186/1532-429X-17-S1-P304

**Published:** 2015-02-03

**Authors:** Enver Tahir, Marc Regier, Martin R Sinn, Dennis Säring, Ulf K Radunski, Gerhard Adam, Gunnar Lund

**Affiliations:** 1 Diagnostic and Interventional Radiology, University Medical Center Hamburg-Eppendorf, Hamburg, Germany; 2 Institute for Computational Neuroscience, University Medical Center Hamburg-Eppendorf, Hamburg, Germany; 3 General and Interventional Cardiology, University Medical Center Hamburg-Eppendorf, Hamburg, Germany

## Background

Regular physical activity over a long time period leads to a cardiac adaptation described as "athlete's heart". The purpose of this study was to determine the effects of intensive daily training in a specific type of sports- professional soccer, in regard to morphological and functional left ventricular parameters assessed by cardiac magnetic resonance imaging (CMRI) and to compare these with non-athletic healthy volunteers.

## Methods

CMRI was performed in 17 male professional soccer players from the German Bundesliga team squad of the Hamburger SV and 8 age-, sex- and weight-matched untrained controls at 1.5 T (Achieva, Philips) during the active season. For quantitative CMRI, an electrocardiographically triggered steady-state free precession (SSFP) cine sequence (TR/TE, 3.2/1.6ms; pixel-size, 1.7mm×1.7mm) was performed in short- and long-axis views. Quantitative analysis included end-diastolic (EDV) and end-systolic volumes (ESV), stroke volume (SV), left ventricular ejection-fraction (EF) as well as end-diastolic (EDMM) and end-systolic myocardial mass (ESMM). CMRI data were analyzed by two independent observers using the HeAT-Software. Data are given as the mean of both observers.

## Results

In professional soccer players a significant increase of the following parameters was determined compared to non-athletes: EDV (229 ±24 ml vs. 196 ±30 ml, *P*< 0.04), ESV (96 ±16 ml vs*.* 82 ±11 ml, *P*< 0.04) and LV mass (189 ±34 g vs. 143 ±19 g, *P*= 0.001). Stroke volume (133 ±19 ml vs. 115 ±23 ml, *P*= ns) and LV ejection fraction (0.58% vs. 0.58%, *P*= ns) were similar in both groups. The professional soccer players had a significantly lower resting heart rate than non-athletes (50 beat/min vs. 64 beat/min, *P*= 0.01).

## Conclusions

Long-term training in professional soccer players is characterised by left ventricular adaptation leading to an increase in functional parameters and myocardial mass. CMRI allows an objective quantitative assessment and might help to differentiate physiologic cardiac adaptations from conditions such as hypertrophic cardiomyopathy.

## Funding

N/A.

**Figure 1:**
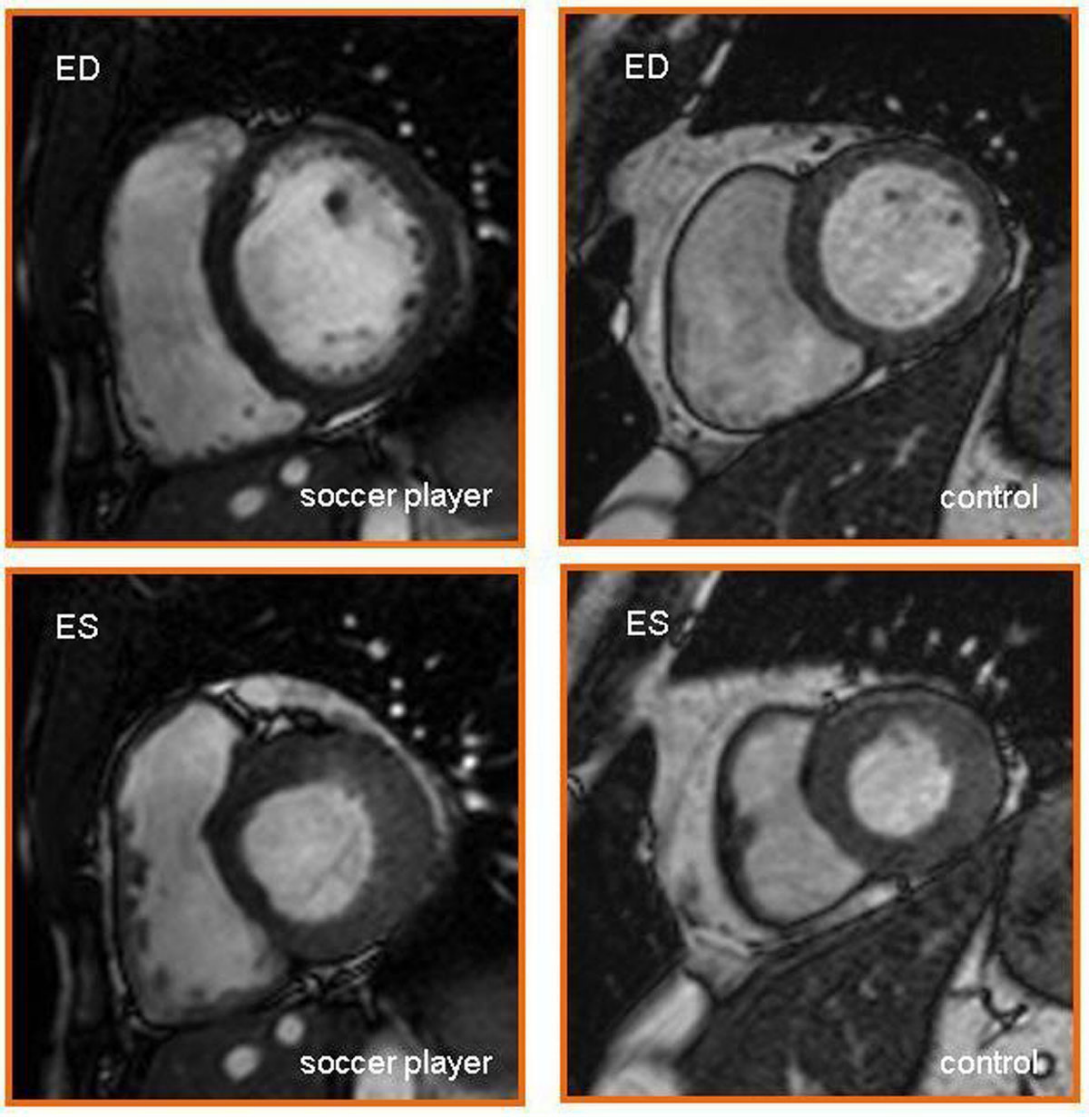
**CMR studies in short-axis view in a soccer player (left) and a control subject (right).** End-diastolic (upper panel) and end-systolic (lower panel) images are depicted. Soccer player's heart shows larger end-diastolic volume and thicker myocardium during systole.

